# Depletion of PARP10 inhibits the growth and metastatic potential of oral squamous cell carcinoma

**DOI:** 10.3389/fgene.2022.1035638

**Published:** 2022-10-13

**Authors:** Zihui Zhou, Bing Wei, Yu Liu, Tian Liu, Sien Zeng, Jinfeng Gan, Guangying Qi

**Affiliations:** ^1^ Guangxi Key Laboratory of Tumor Immunology and Microenvironmental Regulation, Guilin Medical University, Guilin, China; ^2^ Department of Pathology, Affiliated Hospital, Guilin Medical University, Guilin, China

**Keywords:** oral squamous cell carcinoma, PARP10, growth, metastatic potential, PI3K-Akt signaling, MAPK signaling

## Abstract

**Background:** Although poly (ADP-ribose) polymerase family member 10 (PARP10) has been implicated in the progression of multiple cancer types, its role in oral squamous cell carcinoma (OSCC) remains unknown. This study aimed to examine the function of PARP10 in OSCC and investigate the underlying mechanisms.

**Methods:** The expression of PARP10 in OSCC was investigated in OSCC patient cohorts. Kaplan-Meier curve analysis was performed to assess the association between PARP10 and prognosis in OSCC. Correlation between PARP10 expression and the related variables was analyzed by χ^2^ test. CKK-8, transwell assay, western blot, immunohistochemistry, immunofluorescence, and bioinformatic analysis, were applied to clarify the role of PARP10 in OSCC.

**Results:** PARP10 was found to be markedly elevated in OSCC tissues. The upregulation of PARP10 predicted shorter overall survival and disease-specific survival and was significantly correlated with several malignant features. Moreover, depletion of PARP10 markedly inhibited the proliferation, migration, and invasion of OSCC cells, and promoted OSCC cell apoptosis, and resulted in alterations of relevant proteins. Furthermore, a positive correlation was observed between the expression of PARP10 and Ki67, PARP1, MMP2, and VEGF. In addition, depletion of PARP10 impaired the PI3K-AKT and MAPK signaling pathways.

**Conclusion:** PARP10 is involved in the progression of OSCC *via* regulation of PI3K-AKT and MAPK signaling pathways.

## Introduction

Oral squamous cell carcinoma (OSCC) is a commonly occurring head and neck cancer. More than 90% of cancer cases in head and neck region are OSCCs (Quan et al., 2012). Although the management of OSCC has improved, about 50% of patients died within 5 years (Mascitti et al., 2018). Therefore, it is urgent to explore the underlying mechanism of OSCC progression and identify therapeutic targets for improving its clinical treatment.

The poly (ADP ribose) polymerase (PARP) family consists of 17 members characterized by a PARP catalytic domain in the C-terminus (Curtin & Szabo, 2020). PARPs participate in protein posttranslational modifications by transferring multiple ADP-ribose units or mono ADP-ribose units to substrates (Curtin & Szabo, 2020). PARPs are involved in various biological processes (Curtin & Szabo, 2020), including cells proliferation (Schiewer et al., 2012; Choi et al., 2017), apoptosis (Yu et al., 2002; Todorova et al., 2014), inflammation (Kamboj et al., 2013; Rom et al., 2016), DNA damage response (Muller et al., 2019; Wang et al., 2019), gene transcription (Chen et al., 2014; Gibson et al., 2016) and chromatin remodeling (Wright et al., 2016; Hanzlikova et al., 2017).

PARP10, a mono-ADP-ribosyltransferase (Kleine et al., 2008), was initially discovered as an interacting protein of c-Myc (Yu et al., 2005). PARP10 was found to inhibit the transformation of rat embryonic fibroblasts mediated by c-Myc and E1A (Yu et al., 2005). Subsequent studies showed that PARP10 can function as either a tumor suppressor or an oncogene (Schleicher et al., 2018; Zhao et al., 2018; Tian L. et al, 2020). A deficiency of PARP10 promotes the migration and invasion of HeLa cells and hepatocellular carcinoma (HCC) QGY-7703 cells *in vitro* and enhances the *in vivo* metastasis of these cells in a mouse model (Zhao et al., 2018). Recently, a report showed that PARP10 significantly decreases the colony-forming number of HCC cells (Tian L. et al, 2020). In two other reports, the loss of PARP10 was found to reduce the growth of HeLa cells both *in vitro* and *in vivo* (Schleicher et al., 2018) and decreases the proliferation of breast cancer and colorectal adenocarcinoma cells (Marton et al., 2018). In addition, forced expression of PARP10 is sufficient to induce tumor formation by non-transforming RPE-1 cells (Schleicher et al., 2018). Moreover, elevated levels of PARP10 have been observed in up to 19% of breast tumors and 32% of ovarian tumors, as revealed in The Cancer Genome Atlas (TCGA) database (Schleicher et al., 2018). However, the role of PARP10 in OSCC remains unknown.

In the present study, we investigated the clinical significance of PARP10 in OSCC and determined its function and potential mechanisms. We showed that PARP10 was clinically significant in OSCC. Functionally, knockdown of PARP10 inhibited OSCC cell growth, migration, and invasion. Mechanistically, knockdown of PARP10 impaired the PI3K-AKT and MAPK signaling pathways. Thus, these findings suggest that PARP10 functions as an oncogene in OSCC and may be a potential target for OSCC treatment.

## Materials and methods

### Cell culture

The human OSCC cell lines (KB) were obtained from the American Type Culture Collection (ATCC, Manassas, United States). HSC3 and HSC4 cells of OSCC were obtained from Japanese Collection of Research Bioresources Cell Bank. These cells were cultured in Dulbecco’s modified Eagle’s medium (DMEM; Gibco, United States) (KB cells) or RPMI-1640 (Thermo Fisher Scientific, United States) (HSC3 and HSC4 cells) supplemented with 10% fetal bovine serum (Gibco, United States) and 1% penicillin-streptomycin (Solarbio, Beijing, China). All cell cultures were maintained in a humidified incubator at 37°C with 5% CO_2_.

## Patients and clinical samples

Ninety-two surgical OSCC specimens were collected from the Affiliated Hospital of Guilin Medical College from 2010 to 2015. None of the patients received radiotherapy or chemotherapy treatment prior to surgery. All of the patients were diagnosed and confirmed histologically as OSCC. Eleven normal oral epithelium specimens were also collected from the Affiliated Hospital of Guilin Medical College. The study protocol was reviewed and approved by Ethics Committee of Guilin Medical University. Written informed consent was obtained from patients and the study was performed following the Declaration of Helsinki.

### Immunohistochemical (IHC) staining

IHC was performed as previously described (Qi et al., 2010). Briefly, the paraffin-embedded tissues were cut into 4 μm thick slices and then dewaxed and rehydrated. After the endogenous peroxidase was blocked and performing antigen retrieval, the slides were incubated with the primary antibodies against PARP10 (Abcam, Cat. No.ab70800, Ki67 (Dako, Cat. No. M7240), PARP1 (Abcam, Cat. No.ab227244), MMP2 (Abcam, Cat. No.ab86607), or VEGF (Abcam, Cat. No. ab155288) at 4°C overnight, followed by incubation with the horseradish peroxidase-conjugated secondary antibody. Immunostaining signals were detected by 3,3′-diaminobenzidine (DAB) staining, and the nuclei were stained with Harris hematoxylin. The specimens were independently scored by two pathologists who were unaware of the clinical information of these patients. Based on the proportion of positively stained cells, the specimens were classified into four grades: 0–5% (0), 6–20% (1), 21–50% (2), and 51–100% (3). The expression levels of the indicated proteins were divided into two groups based on the proportion score: 0–1 was defined as the low expression group, and 2–3 was defined as the high expression group.

### Small interfering RNA (siRNA) transfection

The cells were seeded into six-well plates and transfected with PARP10 siRNAs or nonspecific siRNA using Lipofectamine 3000 (Thermo Fisher Scientific, United States) according to the manufacturer’s instructions. The siRNA oligonucleotides targeting PARP10 were as follows: siPARP10 #1 sense: 5′-GCA​UCU​GCC​AGC​CCA​GCA​UTT-3′, siPARP10 #2 sense: 5′-GCU​CUA​CCA​UGA​GGA​CCU​UTT-3′ and siPARP10 #3 sense: 5′-CCG​GUC​ACU​GGA​GCC​UCA​ATT-3’. The nonspecific siRNA (sense: 5′-UUC​UCC​GAA​CGU​GUC​ACG​UTT-3′) was used as the negative control.

### Western blot analysis

Western blot was performed as previously described (Qi et al., 2010). Briefly, the cell lysates were collected and subjected to protein extraction with RIPA lysis buffer (Beyotime, Shanghai, China). Then, the total proteins were separated by sodium dodecyl sulfate-polyacrylamide gel electrophoresis (SDS-PAGE) and transferred to polyvinylidene difluoride (PVDF) membranes (Solarbio, Beijing, China). The membranes were blocked with 5% milk, followed by incubation with the primary antibodies against PARP10 (Abcam, Cat. No. ab70800), Cyclin B (Abcam, Cat. No.ab181593), Cyclin D1 (Abcam, Cat. No. ab6152), p53 (Proteintech, Cat. No.60283-1-Ig), p21 (Proteintech, Cat. No.60214-1-Ig), p27 (Proteintech, Cat. No.25614-1-AP), Cleaved Caspase 3 (Cell Signaling Technology Cat. No.9661), Caspase 3 (Proteintech, Cat. No.19677-1-AP), Bax (Proteintech, Cat. No.60267-1-Ig), Bcl2 (Proteintech, Cat. No.60178-1-Ig), Bcl-xL (Proteintech, Cat. No.66020-1-Ig), E-cadherin (Cell Signaling Technology, Cat. No.3195), N-cadherin (Abcam, Cat. No. ab76011), Vimentin (Cell Signaling Technology, Cat. No.5741), Snail (Cell Signaling Technology, Cat. No.3895), Slug (Cell Signaling Technology, Cat. No. 9585), MMP2 (Abcam, Cat. No.ab86607), VEGF (Abcam, Cat. No. ab155288), β-catenin (Cell Signaling Technology, Cat. No.9562), Wnt5a/b (Proteintech, Cat. No.55184-1-AP), p-AKT (Proteintech, Cat. No.66444-1-Ig), AKT (Proteintech, Cat. No.60203-2-Ig), p-SRC (Cell Signaling Technology, Cat. No.2101), SRC (Cell Signaling Technology, Cat. No.2108), p-P38 (Cell Signaling Technology, Cat. No.9216), P38 (Cell Signaling Technology, Cat. No.9212), p-RSK (Cell Signaling Technology, Cat. No.9341), RSK (Cell Signaling Technology, Cat. No.9333), or β-actin (Cat. No.TA-09) overnight at 4°C. After washing with TBST, the membranes were incubated with a secondary antibody for 2 h at room temperature. The immunoblots were visualized by a chemiluminescence reagent (Thermo Fisher). β-actin was used as an internal control.

### Cell proliferation assay

The cells were seeded into 96-well plates at a density of 1,500 cells per well. Ten microliters of CCK-8 (Dojindo, Shanghai, China) solution was added to each well of each group at serial time points: 0 h, 24 h, 48 h, 72 h, and 96 h. After 4 h of incubation with CCK-8, the absorbance was measured at a wavelength of 450 nm.

### Cell migration and invasion assays

The cells (5 × 10^4^) in 200 μL of serum-free RPMI-1640 medium were seeded into the upper Transwell chambers (Corning) without (migration assay) or pre-coated with (invasion assay) Matrigel (BD Bioscience). The lower chambers were filled with 500 μL of RPMI-1640 medium containing 10% FBS. After incubating for 48 h, the migrated/invaded cells were fixed with 4% paraformaldehyde, stained with crystal violet, and counted in five random visual fields per filter under an inverted microscope (IXTIFL, Olympus, Japan).

### Immunofluorescence

The cells grown on the slides were fixed with 4% paraformaldehyde and incubated with 0.5% Triton X-100. Then, the cells were further incubated with an anti-α-tubulin antibody (Abcam, Cat. No. ab52866) for 1 h at room temperature, followed by the secondary antibody for 30 min at room temperature. The nuclei were stained with 1 μg/ml DAPI (Solarbio) for 5 min. Images were captured using a Leica DM3000a (Leica Microsystems Inc, Wetzlar, Germany).

### Data mining

An OSCC dataset GSE37991 (Chou et al., 2019) was downloaded from Gene Expression Omnibus (GEO; https://www.ncbi.nlm.nih.gov/geo/) (Clough & Barrett, 2016), and the expression levels of the PARP family genes were investigated in this dataset, and the differentially expressed genes were ranked by the R package “ggplot2”. The expression of PARP10 in head and neck squamous cell carcinoma (HNSCC) was analyzed in the UALCAN database (http://ualcan.path.uab.edu/index.html) (Chandrashekar et al., 2017). The DNA copy number and amplified status of PARP10 were analyzed in the Oncomine (https://www.oncomine.org/) (Rhodes et al., 2007) and cBioPortal databases (http://www.cbioportal.org/) (Gao et al., 2013), respectively. The protein level of PARP10 in various human tissues and cells was investigated in ProteomicsDB (https://www.proteomicsdb.org/) (Schmidt et al., 2018; Samaras et al., 2020).

### Survival analysis

An OSCC GEO dataset GSE41613 (Lohavanichbutr et al., 2013) containing follow-up data was obtained from GEO. The cohort included 97 OSCC patients with 51 dead patients and 46 alive patients. Among 51 deaths, 30 were OSCC-specific deaths. The probe 238083_at was used to extract the expression data of PARP10, and the cutoff values for PARP10 expression for the OSCC patients’ overall survival and disease-specific survival were determined by receiver operating characteristic (ROC) curves. Based on the cutoff values, the OSCC patients were divided into high and low PARP10 groups. The impact of PARP10 expression on the overall survival and disease-specific survival of OSCC patients was analyzed by Kaplan-Meier curve analysis.

### Gene set enrichment analysis (GSEA)

GSEA was performed in the GSE41613 dataset to analyze the relationship between PARP10 expression and a proliferation signature (BENPORATH_PROLIFERATION), an epithelial-mesenchymal transition (EMT) signature (SARRIO_EPITHELIAL_MESENCHYMAL_TRANSITION_UP) and a glycolysis signature (HALLMARK_GLYCOLYSIS) using GSEA software (http://www.broadinstitue.org/gsea/index.jsp).

### Gene ontology (GO) and KEGG pathway enrichment analysis

The core enrichment genes of HALLMARK_GLYCOLYSIS gene set revealed by GSEA were subject to DAVID database (https://david.ncifcrf.gov/) for GO biological process and Kyoto Encyclopedia of Genes and Genomes (KEGG) pathway enrichments. The differentially expressed genes (DEGs) between *PARP10* ^High^ and *PARP10* ^Low^ patients in the GSE41613 dataset were identified by the “limma” R package. Genes with |Log_2_ (fold change)| > 0.585 and adjusted *p-*value < 0.05 were considered statistically significant. The distribution of DEGs was shown in volcano plots. The upregulated DEGs were subjected to the DAVID database for KEGG pathway enrichment analysis. The enrichment pathways were visualized by the “ggplot2” R package.

### Statistical analysis

All statistical analyses were carried out with SPSS22.0 or GraphPad Prism 8.0. ROC curve was used to assess the accuracy of PARP10 expression in distinguishing OSCC tissues from adjacent nontumor tissues. The χ2 test was used to assess the difference in PARP10 expression between the OSCC and normal oral epithelium tissues, as well as the correlation between PARP10 expression and various variables. Student’s t-test was used to compare the differences between the two selected groups. A *p*-value < 0.05 was considered to be statistically significant.

## Results

### PARP10 is highly expressed in OSCC

**FIGURE 1:**
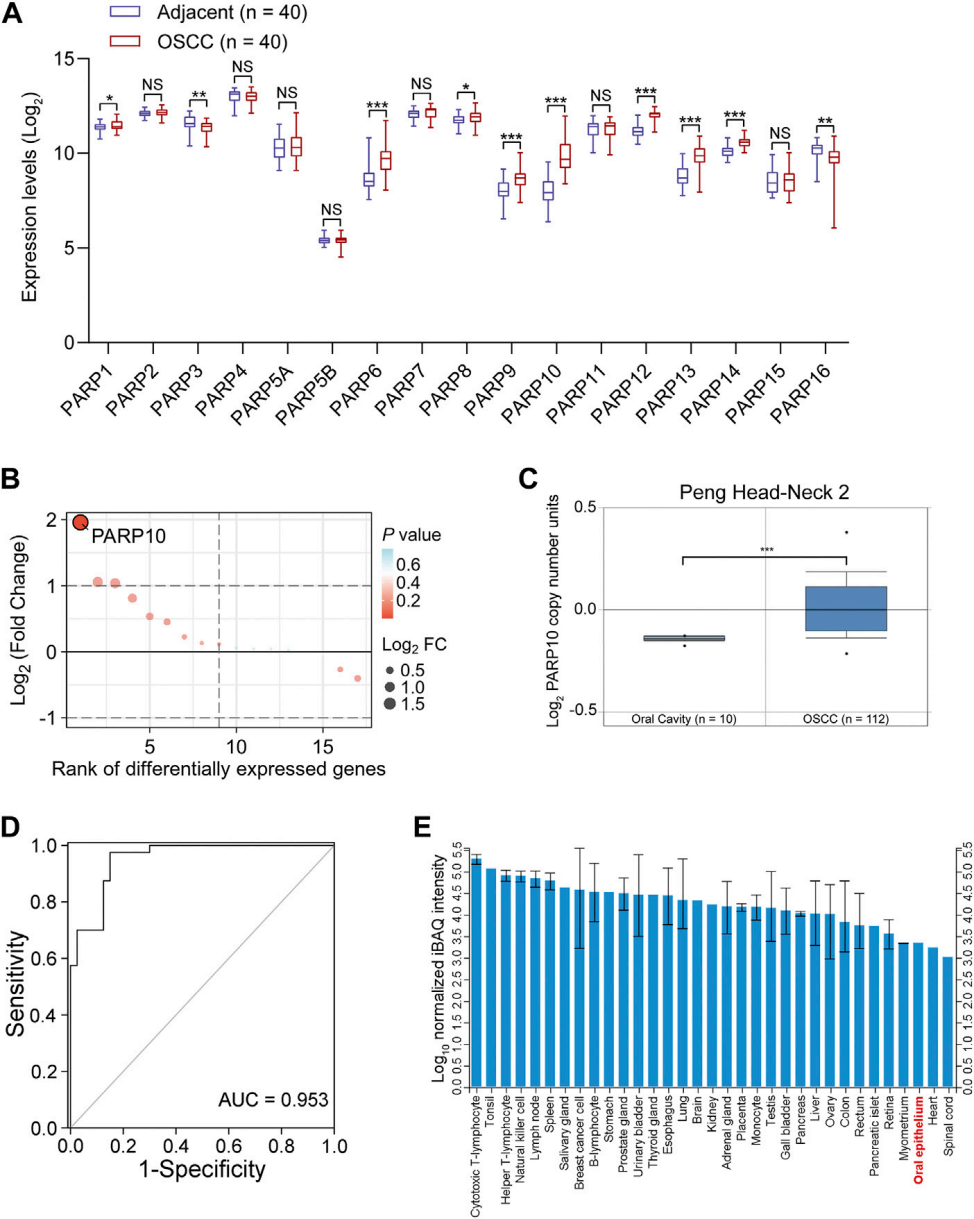
PARP10 is elevated in OSCC. **(A)** The mRNA levels of PARP family genes were investigated in the GEO OSCC dataset GSE37991. **(B)** The differentially expressed PARP family genes were ranked by the R package “ggplot2”. **(C)** The DNA copy number of the PARP10 gene in OSCC was investigated in the Oncomine database. **(D)** ROC analysis was performed to assess the accuracy of PARP10 expression in distinguishing OSCC tissues from adjacent nontumor tissues in GSE37991 cohort. **(E)** The protein level of PARP10 in various human tissues and cells, including oral epithelium, was investigated in ProteomicsDB. NS, not significant. ^*^ *p* < 0.05, ^**^ *p* < 0.01, ^***^ *p* < 0.001.

To better understand the expression of PARP family genes in OSCC, the expression profiles of 17 PARP family genes were first investigated in 40 OSCC tissues and paired adjacent nontumor tissues in GEO OSCC dataset GSE37991. The results revealed that eight genes (PARP1, PARP6, PARP8, PARP9, PARP10, PARP12, PARP13, and PARP14) were upregulated, while two genes (PARP3 and PARP16) were downregulated in OSCC tissues compared to adjacent nontumor tissues (Figure 1A). Further investigation revealed that the most upregulated gene was PARP10 (Figure 1B). Similarly, higher expression of PARP10 was found in head and neck squamous cell carcinoma (HNSCC) (Supplementary Figure S1A, *p* < 0.001). Notably, its expression gradually increased with the advancement of tumor grade (Supplementary Figure S1B) and nodal metastasis (Supplementary Figure S1C). Intriguingly, the expression of PARP10 was more robust in human papillomavirus (HPV)-positive patients than in HPV-negative patients (Supplementary Figure S1D). In addition, we observed that the PARP10 gene was amplified in OSCC (Figure 1C) and HNSCC (Supplementary Figure S2). ROC analysis revealed that PARP10 could distinguish OSCC tissues from adjacent nontumor tissues [Figure 1D; Supplementary Table S1, area under curve (AUC) = 0.953, sensitivity = 97.5%, specificity = 85.0%, positive predictive value = 0.867, negative predictive value = 0.971]. Furthermore, when compared to other human tissues, the expression of PARP10 was found to be lower in oral epithelium (Figure 1E). These findings suggest that PARP10 is upregulated in OSCC.

### Upregulation of PARP10 is associated with an adverse prognosis in OSCC

**FIGURE 2:**
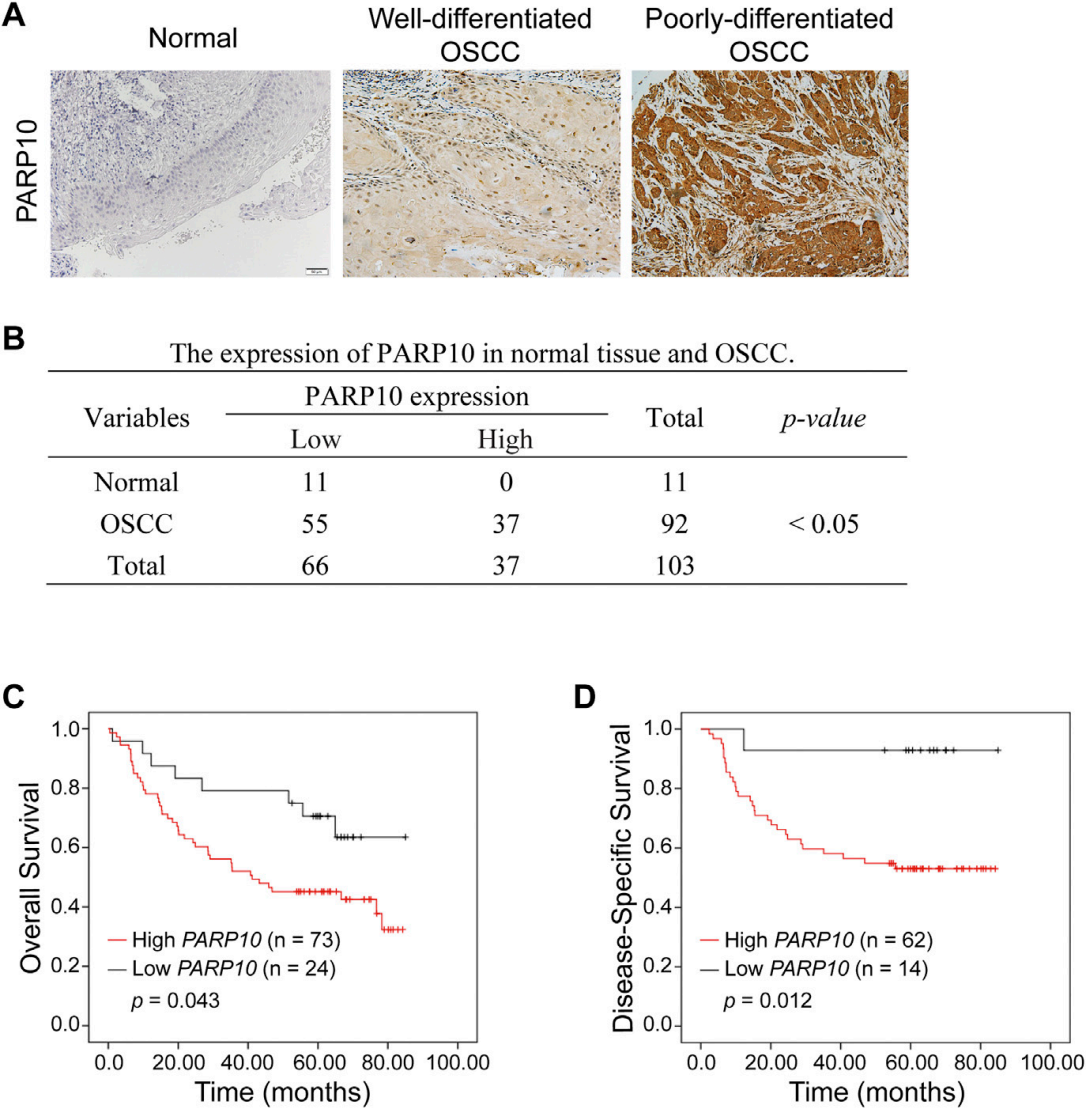
Upregulation of PARP10 is associated with poor survival in OSCC patients. **(A,B)** The protein levels of PARP10 in OSCC and normal oral epithelium tissues were investigated by immunohistochemistry **(A)**, and the difference in PARP10 expression between the two groups was analyzed using the χ2 test **(B)**. **(C,D)** Kaplan-Meier analysis was performed to assess the association between *PARP10* expression and the overall survival **(C)** and disease-specific survival **(D)** of OSCC patients in the GSE41613 dataset.

The above observations were further verified in our patient cohort by immunohistochemistry (Figure 2A). PARP10 protein expression was remarkably higher in OSCC than in normal oral epithelium tissues (Figure 2B, *p* < 0.05). PARP10 protein was expressed in high levels in 37 of 92 patients, while 55 of 92 patients had a low expression level of PARP10 (Figure 2B). In contrast, PARP10 protein expression was low in 11 of 11 normal oral epithelium tissues (Figure 2B). Correlation analysis showed that high PARP10 expression was significantly associated with several malignant features, including histological differentiation (Table 1, *p* < 0.001), lymph node metastasis (Table 1, *p* < 0.05) and clinical stage (Table 1, *p* < 0.01). We further assessed the prognostic value of PARP10 expression in the OSCC dataset GSE41613 with survival data. As shown in Figures 2C,D; Supplementary Figure S3, OSCC patients with high *PARP10* expression had shorter overall survival (*p* = 0.043) and disease-specific survival (*p* = 0.012) than those with low *PARP10* expression.

**TABLE 1:** The correlation between PARP10 expression and clinicopathological features in OSCC.

Clinicopathological features	No. of patients	PARP10 expression		*p-value*
		Low, *n* (%)	High, *n* (%)	
All patients	92	55 (59.8)	37 (40.2)	
Age				
≥57	54	35 (64.8)	19 (35.2)	>0.05
<57	38	20 (52.6)	18 (47.4)	
Sex				
Male	66	37 (56.1)	29 (43.9)	>0.05
Female	26	18 (69.2)	8 (30.8)	
Histological differentiation				
Well/Moderate	69	49 (71.0)	20 (29.0)	<0.001
Poor	23	6 (26.1)	17 (73.9)	
Lymph node metastasis				
Negative	53	37 (69.8)	16 (30.2)	<0.05
Positive	39	18 (46.2)	21 (53.8)	
Clinical stage				
Ⅰ	56	40 (71.4)	16 (28.6)	<0.01
Ⅱ/Ⅲ	36	15 (41.7)	21 (58.3)	

PARP10, poly (ADP-ribose) polymerase family member 10; OSCC, oral squamous cell carcinoma.

### PARP10 is linked to various malignant biological processes

**FIGURE 3:**
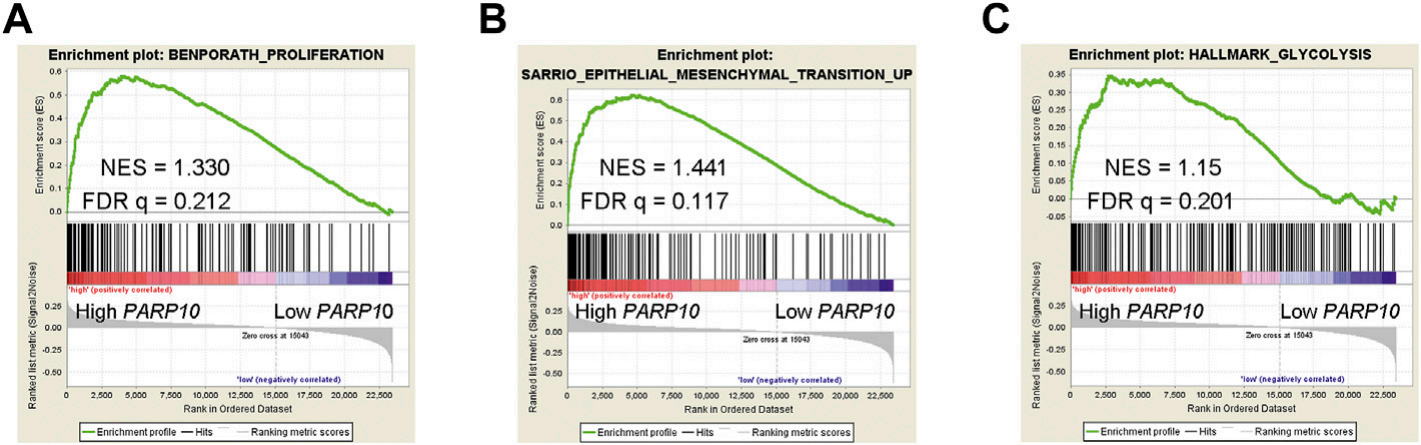
PARP10 is positively associated with proliferation, EMT, and glycolysis signatures. **(A)** GSEA plots of enrichment of BENPORATH_PROLIFERATION signature in *PARP10* ^High^ tumors *versus PARP10* ^Low^ tumors in the GSE41613 dataset. **(B)** GSEA plots of enrichment of SARRIO_EPITHELIAL_MESENCHYMAL_TRANSITION_UP signature in *PARP10* ^High^ tumors *versus PARP10* ^Low^ tumors in the GSE41613 dataset. **(C)** GSEA plots of enrichment of HALLMARK_GLYCOLYSIS signature in *PARP10* ^High^ tumors *versus PARP10* ^Low^ tumors in the GSE41613 dataset.

The clinical significance of PARP10 in OSCC suggests that PARP10 may play a role in OSCC progression. To elucidate the role of PARP10 in OSCC, we applied gene set enrichment analysis (GSEA) to investigate the malignant behaviors in which PARP10 may be involved. The results showed that the proliferation signature (BENPORATH_PROLIFERATION) [Figure 3A, normalized enrichment score (NES) = 1.330, false discovery rate (FDR) q = 0.212], epithelial-mesenchymal transition (EMT) signature SARRIO_EPITHELIAL_MESENCHYMAL_TRANSITION_UP (Figure 3B, NES = 1.441, FDR q = 0.117) were enriched in the OSCC patients with high *PARP10* expression. Intriguingly, glycolysis signature HALLMARK_GLYCOLYSIS was also found to be enriched in OSCC patients with high *PARP10* expression (Figure 3C, NES = 1.15, FDR q = 0.201). Further, the core enrichment genes of HALLMARK_GLYCOLYSIS gene set (Table S2) were subject to GO analysis and found that these genes mainly regulated various metabolic processes including gluconeogenesis, carbohydrate metabolic process, galactose catabolic process, galactose metabolic process, fructose 1,6-bisphosphate metabolic process, pyruvate metabolic process, and canonical glycolysis (Supplementary Figure S4A). KEGG pathway enrichment analysis showed that these genes were mainly enriched in the HIF-1 signaling pathway, biosynthesis of amino acids, metabolic pathways, glycolysis/gluconeogenesis, galactose metabolism, carbon metabolism, and central carbon metabolism in cancer (Supplementary Figure S4B). These results suggest a critical role of PARP10 in OSCC.

### Depletion of PARP10 represses growth and metastatic capability in OSCC cells

**FIGURE 4:**
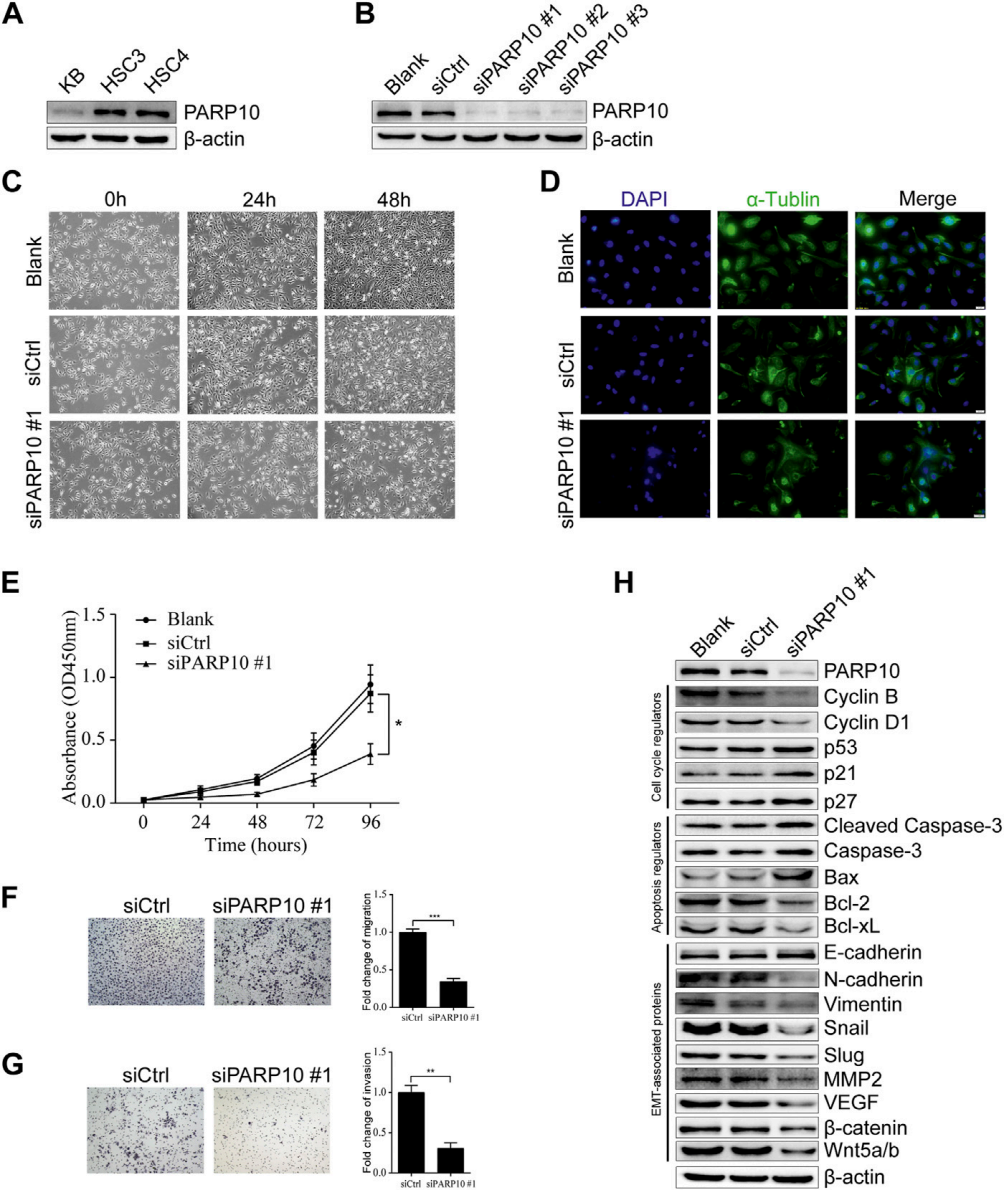
Depletion of PARP10 represses growth and metastatic capability in OSCC cells. **(A)** Western blot analysis of PARP10 in OSCC cell lines. β-actin was used as an internal control. **(B)** Western blot analysis of PARP10 in HSC3 cells transfected with siRNAs targeting PARP10. β-actin was used as an internal control. **(C)** Morphological images of HSC3 cells treated with PARP10 siRNA at the indicated time points. **(D)** Immunofluorescence assay of α-tubulin (green) in HSC3 cells transfected with PARP10 siRNA. Nuclei were stained with DAPI (blue). **(E)** The proliferation of HSC3 cells transfected with PARP10 siRNA was monitored by CCK-8 assays at the indicated time points. **(F)** Migration assay of HSC3 cells transfected with PARP10 siRNA (left). Fold change of migration was quantified (Right). **(G)** Invasion assay of HSC3 cells transfected with PARP10 siRNA (left). Fold change of invasion was quantified (Right). **(H)** Western blot analysis of the cell cycle regulators, apoptosis-related proteins, and EMT-related proteins in HSC3 cells transfected with PARP10 siRNA. β-actin was used as an internal control. ^*^ *p* < 0.05, ^**^ *p* < 0.01, ^***^ *p* < 0.001.

Based on the above observations, we next evaluated the effect of PARP10 on OSCC cell growth. First, we detected the expression of PARP10 in OSCC cell lines and found that PARP10 was differentially expressed in three OSCC cell lines (Figure 4A). HSC3 was used as a cell model for the subsequent experiments. The siRNA oligonucleotides targeting PARP10 were applied to silence the expression of PARP10 in HSC3 cells, and all three siRNA oligonucleotides remarkably downregulated the expression of PARP10 (Figure 4B). PARP10 siRNA #1 was selected for the subsequent experiments. The PARP10 siRNA strongly decreased the HSC3 cell numbers (Figure 4C) as revealed by optical microscopy, and some cells exhibited distinct morphology characterized by nuclear atypia (Figure 4D), which resembled apoptosis. Consistently, silencing PARP10 significantly reduced HSC3 cell proliferation (Figure 4E, *p* < 0.05). In support, silencing PARP10 decreased cell cycle accelerators (Cyclin B and Cyclin D1) and increased the expression of cell cycle inhibitors (p53, p21, and p27) (Figure 4H). Moreover, silencing PARP10 increased the expression of apoptosis-promoting proteins (Cleaved Caspase-3, Caspase-3 and Bax) and decreased the expression of apoptosis-inhibiting proteins (Bcl-2 and Bcl-xL) (Figure 4H).

Further, we examined the effect of PARP10 deficiency on OSCC cell metastatic potential. The results showed that silencing of PARP10 dramatically reduced both the migratory (Figure 4F, *p* < 0.001) and invasive (Figure 4G, *p* < 0.01) capabilities of HSC3 cells. These data indicate that PARP10 may play an important role in the promotion of OSCC cell migration and invasion. As it is well established that epithelial-mesenchymal transition (EMT) plays an indispensable role in tumor invasion and metastasis (Pastushenko & Blanpain, 2019), we next detected the expression levels of EMT-associated proteins by western blot. The results showed that silencing PARP10 slightly induced the expression level of the epithelial marker E-cadherin, but diminished the expression levels of mesenchymal markers (N-cadherin and Vimentin) and the expression levels of EMT activators (Snail, Slug, MMP2, VEGF, β-catenin, and Wnt5a/b) in HSC3 cells (Figure 4H). Taken together, these results suggest that PARP10 promotes OSCC growth *via* regulation of the cell cycle and apoptosis signaling and that PARP10 enhances OSCC cell migration and invasion through regulation of EMT.

### PARP10 is correlated with multiple oncogenic proteins in OSCC tissues

**FIGURE 5:**
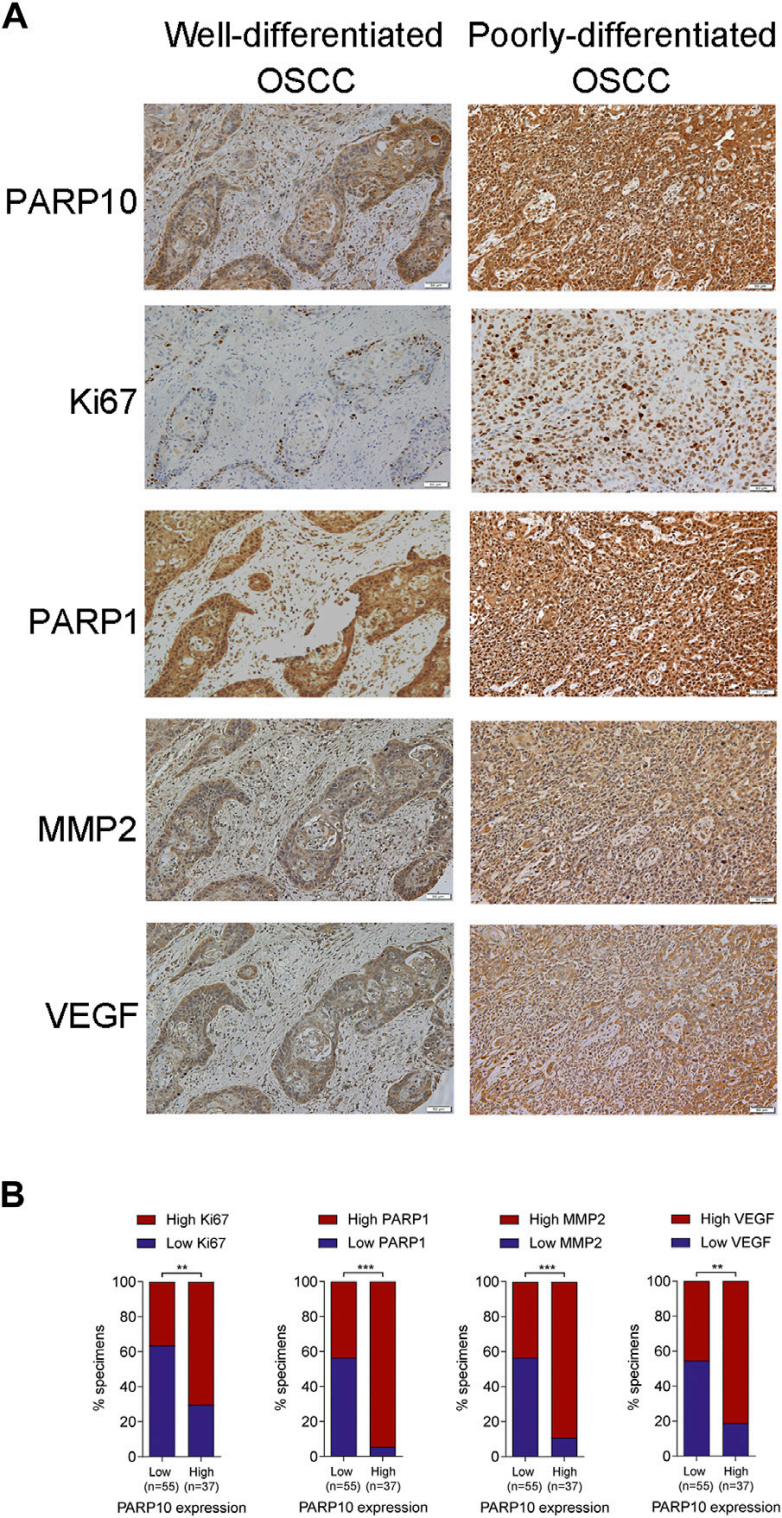
PARP10 is positively correlated with Ki67, PARP1, MMP2, and VEGF in OSCC tissues. **(A)** Representative immunohistochemistry images for PARP10, Ki67, PARP1, MMP2, and VEGF in OSCC specimens. **(B)** The correlation between PARP10 expression and the indicated proteins was analyzed using the χ2 test. ^**^ *p* < 0.01, ^***^ *p* < 0.001.

To verify the above results, we compared the expression patterns of PARP10 with well-known proliferation (Ki67), apoptosis (PARP1), and metastasis (VEGF and MMP2)-associated proteins in OSCC tissues. In accordance with the above findings, the expression pattern of PARP10 appeared to be highly consistent with those of Ki67, PARP1, MMP2, and VEGF in OSCC (Figure 5A). Correlation analysis demonstrated a positive relationship between the expression of PARP10 and the expression of Ki67 (Figure 5B, *p* < 0.01), PARP1 (Figure 5B, *p* < 0.001), MMP2 (Figure 5B, *p* < 0.001), and VEGF (Figure 5B, *p* < 0.01). These data further support the role of PARP10 in tumor growth and metastasis of OSCC.

### Silencing of PARP10 impairs the PI3K-AKT and MAPK signaling pathways

**FIGURE 6:**
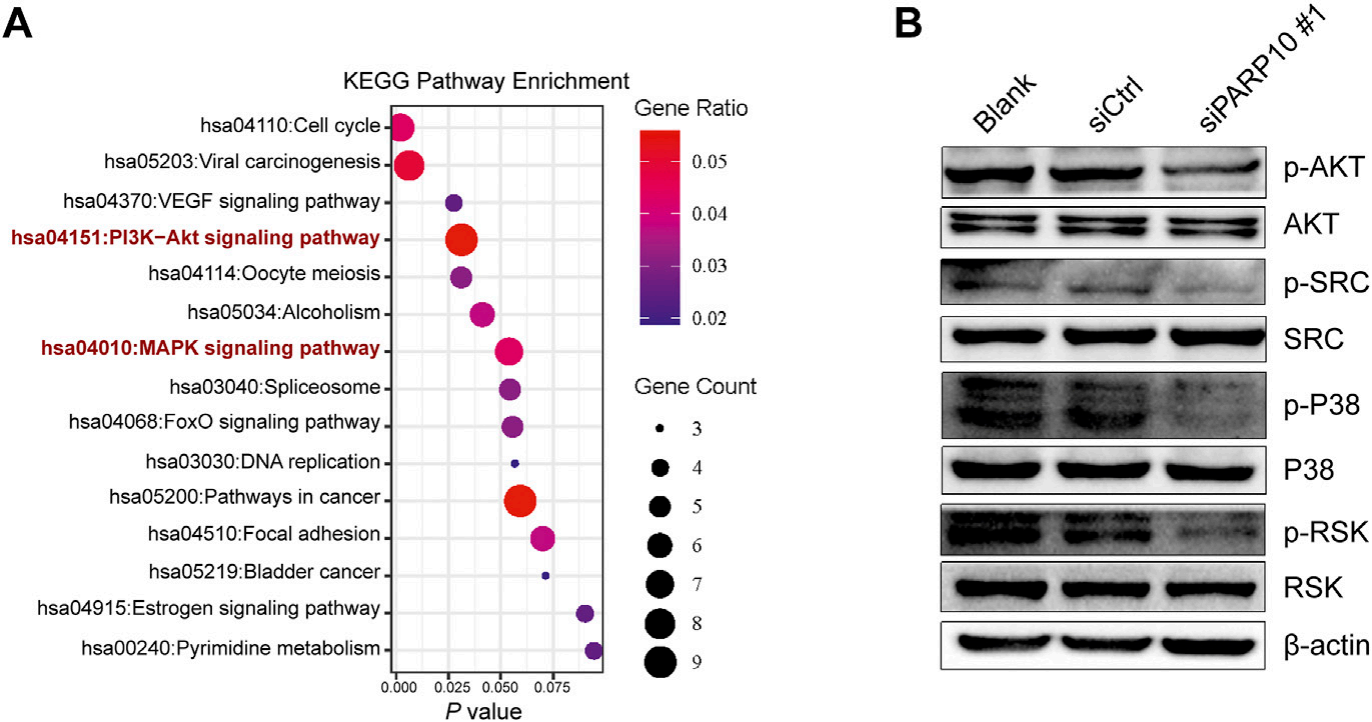
Depletion of PARP10 impairs the PI3K-AKT and MAPK signaling pathways. **(A)** KEGG pathway enrichment analysis of upregulated genes in *PARP10* ^High^ *versus PARP10* ^Low^ OSCC in GSE41613. **(B)** Western blot analysis of proteins that are involved in the PI3K-AKT and MAPK signaling pathways. β-actin was used as an internal control.

We next explored the mechanisms involved in the regulation of OSCC malignant behaviors by PARP10. A total of 161 upregulated genes in *PARP10* ^High^ *versus PARP10* ^Low^ were identified in the OSCC dataset GSE41613 (Supplementary Figure S5). These upregulated genes were subjected to KEGG pathway analysis, and the results showed that these upregulated genes were enriched in multiple pathways, including the PI3K-AKT and MAPK signaling pathways (Figure 6A), which have been demonstrated to contribute to the tumor growth and metastasis of various cancers (Manning & Toker, 2017; Peluso et al., 2019). Therefore, we investigated the impact of PARP10 on these two pathways and found that silencing PARP10 decreased the protein levels of p-AKT, p-SRC, p-P38, and p-RSK in HSC3 cells (Figure 6B). These findings suggest that the tumor-promoting function of PARP10 in OSCC may be at least partially mediated by the PI3K-AKT and MAPK signaling pathways.

## Discussion

Recently, the physiological role of PARP10 in cancers has begun to be understood; however, to date, neither the clinical significance of PARP10 nor the function and downstream of PARP10 in OSCC has been clarified. In this study, we found that PARP10 was upregulated in OSCC and HNSCC and that PARP10 had the potential to differentiate OSCC tissues from adjacent nontumor tissues. Importantly, its upregulation predicted a poor prognosis in OSCC patients. Moreover, we identified the role of PARP10 in OSCC cell proliferation, apoptosis, and metastatic potential. Furthermore, PARP10 was found to be involved in the regulation of the PI3K-AKT and MAPK signaling pathways.

Increased PARP10 expression has been reported in breast and ovarian tumors (Schleicher et al., 2018). Consistently, we found that both the transcription and protein levels of PARP10 were upregulated in OSCC and HNSCC, and that it could efficiently discriminate OSCC tissues from adjacent nontumor tissues, suggesting PARP10 may be useful for diagnosis of OSCC, which needs to be validated in other independent cohorts. Moreover, increased PARP10 protein levels were correlated with several clinicopathologic indicators, including histological differentiation, lymph node metastasis, and clinical stage. The amplification of the PARP10 gene in OSCC and HNSCC tissues and the upregulation of PARP10 expression in HPV-positive HNSCC patients suggest that a gain in gene copy number and HPV infection may at least partially contribute to PARP10 overexpression in OSCC and HNSCC. Given that HPV is a potential risk factor for OSCC (Chai et al., 2020), HPV may contribute to the tumorigenesis of OSCC partially through the upregulation of PARP10. Importantly, elevated PARP10 predicted unfavorable outcomes in OSCC patients. In support, in gastric cancer, high PARP10 expression is correlated with shorter survival (Marton et al., 2018).

PARP10 has been implicated in various biological processes (Feijs et al., 2013; Kaufmann et al., 2015; Marton et al., 2018; Tian Y. et al, 2020; Gao et al., 2020), including the regulation of cancer cell proliferation (Schleicher et al., 2018; Wu et al., 2020) and tumor metastasis (Zhao et al., 2018; Zhao et al., 2021). By performing GSEA, we showed that PARP10 was closely associated with cell proliferation and EMT signatures. Importantly, the experimental data confirmed that PARP10 deficiency markedly inhibited proliferation, migration, and invasion of OSCC cells, and enhanced OSCC cell apoptosis. Moreover, these observations were verified by the detection of proliferation-, apoptosis- and EMT-associated molecules. In addition, we also found that PARP10 was closely correlated with proliferation-, apoptosis-, and metastasis-associated molecules in OSCC tissues. These data further support the role of PARP10 in the regulation of OSCC cell proliferation, apoptosis, and metastatic capability. Besides, we found that PARP10 may be involved in the regulation of glycolysis. In support, PARP10 has been implicated in the regulation of metabolism (Szanto & Bai, 2020). PARP1 and PARP2, two PARP family members, have been demonstrated to be involved in metabolic regulation, including glucose metabolism (Bai & Canto, 2012). In addition, it has been reported that poly-ADP ribose (PAR), can interact with PKM2 (a key glycolytic enzyme), and that this interaction is required for nuclear PKM2-induced glycolysis. PARP inhibitors can eliminate the interaction of PARP/PKM2, thereby suppressing nuclear PKM2-dependent glycolysis and tumor growth (Li et al., 2016). These reports suggest that the PARP family may play a critical role in glycolysis. Whether PARP10 plays a role in the regulation of glycolysis is worth further investigation.

As a mono-ADP-ribosyltransferase, PARP10 has been reported to participate in the regulation of multiple pathways, a role that is dependent on its catalytic activity (Verheugd et al., 2013; Zhao et al., 2018; Tian L. et al, 2020). PARP10 can inhibit Aurora A activity by interacting with it and mono-ADP-ribosylating it, which leads to inhibition of tumor metastasis (Zhao et al., 2018). Recently, a report showed that PARP10 can mono-ADP-ribosylate PLK1, thereby inhibiting its kinase activity and suppressing HCC progression (Tian L. et al, 2020). In another report, PARP10 was demonstrated to perform its oncogenic activity by alleviating replication stress (Schleicher et al., 2018). Moreover, PARP10 has also been revealed to modulate mitochondrial oxidative metabolism *via* regulation of AMPK, thereby affecting cancer cell proliferation (Marton et al., 2018). Here, we showed that two pathways critical for cancer cell proliferation and metastasis, the PI3K-AKT and MAPK signaling pathways, were hampered by silencing PARP10. These data suggest the possibility that PARP10 may regulate the PI3K-AKT and MAPK signaling pathways indirectly or directly. Whether PARP10 activates PI3K-AKT and MAPK signaling pathways *via* its mono-ADP-ribosyltransferase activity or not is worthy of further investigation.

Nonetheless, the results of this study were mainly derived from the depletion of PARP10 in one cell line, and the conclusions need to be verified in cells with PARP10 overexpressed, as well as in more cell lines and mouse models. The clinical significance of PARP10 also needs to be validated in additional independent cohorts. Further investigation of the mechanisms responsible for the function of PARP10 will enhance our understanding of the role of PARP10 in OSCC. Given that a pipeline of inhibitors targeting PARP10 has been developed (Ekblad et al., 2015; Murthy et al., 2018; Morgan et al., 2019; Lemke et al., 2020), it will be important to verify the effects of these inhibitors using OSCC as a model.

In conclusion, the results of this study indicate that PARP10 is upregulated in OSCC, and its overexpression is associated with a poor prognosis. Moreover, PARP10 has exhibited a substantial role in the regulation of OSCC cell proliferation, apoptosis, and metastatic capability. Its effects on the PI3K-AKT and MAPK signaling pathways may explain the function of PARP10 in OSCC. This finding provides a rationale for exploiting PARP10 as a diagnostic, prognostic, and therapeutic target for OSCC.

## Data Availability

The original contributions presented in the study are included in the article/Supplementary Material, further inquiries can be directed to the corresponding authors.

## References

[B1] Bai P. Canto C. (2012). The role of PARP-1 and PARP-2 enzymes in metabolic regulation and disease. Cell Metab. 16 (3), 290–295. 10.1016/j.cmet.2012.06.016 22921416

[B2] Chai A. W. Y. Lim K. P. Cheong S. C. (2020). Translational genomics and recent advances in oral squamous cell carcinoma. Semin. Cancer Biol. 61, 71–83. 10.1016/j.semcancer.2019.09.011 31542510

[B3] Chandrashekar D. S. Bashel B. Balasubramanya S. A. H. Creighton C. J. Ponce-Rodriguez I. Chakravarthi B. (2017). Ualcan: A portal for facilitating tumor subgroup gene expression and survival analyses. Neoplasia 19 (8), 649–658. 10.1016/j.neo.2017.05.002 28732212 PMC5516091

[B4] Chen H. Ruiz P. D. Novikov L. Casill A. D. Park J. W. Gamble M. J. (2014). MacroH2A1.1 and PARP-1 cooperate to regulate transcription by promoting CBP-mediated H2B acetylation. Nat. Struct. Mol. Biol. 21 (11), 981–989. 10.1038/nsmb.2903 25306110 PMC4221384

[B5] Choi J. Xu M. Makowski M. M. Zhang T. Law M. H. Kovacs M. A. (2017). A common intronic variant of PARP1 confers melanoma risk and mediates melanocyte growth via regulation of MITF. Nat. Genet. 49 (9), 1326–1335. 10.1038/ng.3927 28759004

[B6] Chou S. T. Peng H. Y. Mo K. C. Hsu Y. M. Wu G. H. Hsiao J. R. (2019). MicroRNA-486-3p functions as a tumor suppressor in oral cancer by targeting DDR1. J. Exp. Clin. Cancer Res. 38 (1), 281. 10.1186/s13046-019-1283-z 31253192 PMC6599238

[B7] Clough E. Barrett T. (2016). The gene expression Omnibus database. Methods Mol. Biol. 1418, 93–110. 10.1007/978-1-4939-3578-9_5 27008011 PMC4944384

[B8] Curtin N. J. Szabo C. (2020). Poly(ADP-ribose) polymerase inhibition: Past, present and future. Nat. Rev. Drug Discov. 19 (10), 711–736. 10.1038/s41573-020-0076-6 32884152

[B9] Ekblad T. Lindgren A. E. Andersson C. D. Caraballo R. Thorsell A. G. Karlberg T. (2015). Towards small molecule inhibitors of mono-ADP-ribosyltransferases. Eur. J. Med. Chem. 95, 546–551. 10.1016/j.ejmech.2015.03.067 25847771

[B10] Feijs K. L. Verheugd P. Luscher B. (2013). Expanding functions of intracellular resident mono-ADP-ribosylation in cell physiology. FEBS J. 280 (15), 3519–3529. 10.1111/febs.12315 23639026

[B11] Gao J. Aksoy B. A. Dogrusoz U. Dresdner G. Gross B. Sumer S. O. (2013). Integrative analysis of complex cancer genomics and clinical profiles using the cBioPortal. Sci. Signal. 6 (269), pl1. 10.1126/scisignal.2004088 23550210 PMC4160307

[B12] Gao X. Q. Zhang Y. H. Liu F. Ponnusamy M. Zhao X. M. Zhou L. Y. (2020). The piRNA CHAPIR regulates cardiac hypertrophy by controlling METTL3-dependent N(6)-methyladenosine methylation of Parp10 mRNA. Nat. Cell Biol. 22 (11), 1319–1331. 10.1038/s41556-020-0576-y 33020597

[B13] Gibson B. A. Zhang Y. Jiang H. Hussey K. M. Shrimp J. H. Lin H. (2016). Chemical genetic discovery of PARP targets reveals a role for PARP-1 in transcription elongation. Science 353 (6294), 45–50. 10.1126/science.aaf7865 27256882 PMC5540732

[B14] Hanzlikova H. Gittens W. Krejcikova K. Zeng Z. Caldecott K. W. (2017). Overlapping roles for PARP1 and PARP2 in the recruitment of endogenous XRCC1 and PNKP into oxidized chromatin. Nucleic Acids Res. 45 (5), 2546–2557. 10.1093/nar/gkw1246 27965414 PMC5389470

[B15] Kamboj A. Lu P. Cossoy M. B. Stobart J. L. Dolhun B. A. Kauppinen T. M. (2013). Poly(ADP-ribose) polymerase 2 contributes to neuroinflammation and neurological dysfunction in mouse experimental autoimmune encephalomyelitis. J. Neuroinflammation 10, 49. 10.1186/1742-2094-10-49 23607899 PMC3640934

[B16] Kaufmann M. Feijs K. L. Luscher B. (2015). Function and regulation of the mono-ADP-ribosyltransferase ARTD10. Curr. Top. Microbiol. Immunol. 384, 167–188. 10.1007/82_2014_379 24878761

[B17] Kleine H. Poreba E. Lesniewicz K. Hassa P. O. Hottiger M. O. Litchfield D. W. (2008). Substrate-assisted catalysis by PARP10 limits its activity to mono-ADP-ribosylation. Mol. Cell 32 (1), 57–69. 10.1016/j.molcel.2008.08.009 18851833

[B18] Lemke M. Ravenscroft H. Rueb N. J. Kireev D. Ferraris D. Franzini R. M. (2020). Integrating DNA-encoded chemical libraries with virtual combinatorial library screening: Optimizing a PARP10 inhibitor. Bioorg. Med. Chem. Lett. 30 (19), 127464. 10.1016/j.bmcl.2020.127464 32768646 PMC7530011

[B19] Li N. Feng L. Liu H. Wang J. Kasembeli M. Tran M. K. (2016). PARP inhibition suppresses growth of EGFR-mutant cancers by targeting nuclear PKM2. Cell Rep. 15 (4), 843–856. 10.1016/j.celrep.2016.03.070 27149849 PMC5063668

[B20] Lohavanichbutr P. Mendez E. Holsinger F. C. Rue T. C. Zhang Y. Houck J. (2013). A 13-gene signature prognostic of HPV-negative OSCC: Discovery and external validation. Clin. Cancer Res. 19 (5), 1197–1203. 10.1158/1078-0432.CCR-12-2647 23319825 PMC3593802

[B21] Manning B. D. Toker A. (2017). AKT/PKB signaling: Navigating the network. Cell 169 (3), 381–405. 10.1016/j.cell.2017.04.001 28431241 PMC5546324

[B22] Marton J. Fodor T. Nagy L. Vida A. Kis G. Brunyanszki A. (2018). PARP10 (ARTD10) modulates mitochondrial function. PLoS One 13 (1), e0187789. 10.1371/journal.pone.0187789 29293500 PMC5749700

[B23] Mascitti M. Orsini G. Tosco V. Monterubbianesi R. Balercia A. Putignano A. (2018). An overview on current non-invasive diagnostic devices in oral oncology. Front. Physiol. 9, 1510. 10.3389/fphys.2018.01510 30410451 PMC6209963

[B24] Morgan R. K. Kirby I. T. Vermehren-Schmaedick A. Rodriguez K. Cohen M. S. (2019). Rational design of cell-active inhibitors of PARP10. ACS Med. Chem. Lett. 10 (1), 74–79. 10.1021/acsmedchemlett.8b00429 30655950 PMC6331153

[B25] Muller K. H. Hayward R. Rajan R. Whitehead M. Cobb A. M. Ahmad S. (2019). Poly(ADP-Ribose) links the DNA damage response and biomineralization. Cell Rep. 27 (11), 3124–3138. e13. 10.1016/j.celrep.2019.05.038 31189100 PMC6581741

[B26] Murthy S. Desantis J. Verheugd P. Maksimainen M. M. Venkannagari H. Massari S. (2018). 4-(Phenoxy) and 4-(benzyloxy)benzamides as potent and selective inhibitors of mono-ADP-ribosyltransferase PARP10/ARTD10. Eur. J. Med. Chem. 156, 93–102. 10.1016/j.ejmech.2018.06.047 30006177

[B27] Pastushenko I. Blanpain C. (2019). EMT transition states during tumor progression and metastasis. Trends Cell Biol. 29 (3), 212–226. 10.1016/j.tcb.2018.12.001 30594349

[B28] Peluso I. Yarla N. S. Ambra R. Pastore G. Perry G. (2019). MAPK signalling pathway in cancers: Olive products as cancer preventive and therapeutic agents. Semin. Cancer Biol. 56, 185–195. 10.1016/j.semcancer.2017.09.002 28912082

[B29] Qi G. Kudo Y. Ando T. Tsunematsu T. Shimizu N. Siriwardena S. B. (2010). Nuclear Survivin expression is correlated with malignant behaviors of head and neck cancer together with Aurora-B. Oral Oncol. 46 (4), 263–270. 10.1016/j.oraloncology.2010.01.004 20138567

[B30] Quan J. Johnson N. W. Zhou G. Parsons P. G. Boyle G. M. Gao J. (2012). Potential molecular targets for inhibiting bone invasion by oral squamous cell carcinoma: A review of mechanisms. Cancer Metastasis Rev. 31 (1-2), 209–219. 10.1007/s10555-011-9335-7 22101806

[B31] Rhodes D. R. Kalyana-Sundaram S. Mahavisno V. Varambally R. Yu J. Briggs B. B. (2007). Oncomine 3.0: Genes, pathways, and networks in a collection of 18, 000 cancer gene expression profiles. Neoplasia 9 (2), 166–180. 10.1593/neo.07112 17356713 PMC1813932

[B32] Rom S. Zuluaga-Ramirez V. Reichenbach N. L. Dykstra H. Gajghate S. Pacher P. (2016). PARP inhibition in leukocytes diminishes inflammation via effects on integrins/cytoskeleton and protects the blood-brain barrier. J. Neuroinflammation 13 (1), 254. 10.1186/s12974-016-0729-x 27677851 PMC5039899

[B33] Samaras P. Schmidt T. Frejno M. Gessulat S. Reinecke M. Jarzab A. (2020). ProteomicsDB: A multi-omics and multi-organism resource for life science research. Nucleic Acids Res. 48 (D1), D1153–D1163. 10.1093/nar/gkz974 31665479 PMC7145565

[B34] Schiewer M. J. Goodwin J. F. Han S. Brenner J. C. Augello M. A. Dean J. L. (2012). Dual roles of PARP-1 promote cancer growth and progression. Cancer Discov. 2 (12), 1134–1149. 10.1158/2159-8290.CD-12-0120 22993403 PMC3519969

[B35] Schleicher E. M. Galvan A. M. Imamura-Kawasawa Y. Moldovan G. L. Nicolae C. M. (2018). PARP10 promotes cellular proliferation and tumorigenesis by alleviating replication stress. Nucleic Acids Res. 46 (17), 8908–8916. 10.1093/nar/gky658 30032250 PMC6158756

[B36] Schmidt T. Samaras P. Frejno M. Gessulat S. Barnert M. Kienegger H. (2018). ProteomicsDB. Nucleic Acids Res. 46 (D1), D1271–D1281. 10.1093/nar/gkx1029 29106664 PMC5753189

[B37] Szanto M. Bai P. (2020). The role of ADP-ribose metabolism in metabolic regulation, adipose tissue differentiation, and metabolism. Genes Dev. 34 (5-6), 321–340. 10.1101/gad.334284.119 32029456 PMC7050491

[B38] Tian L. Yao K. Liu K. Han B. Dong H. Zhao W. (2020a). PLK1/NF-κB feedforward circuit antagonizes the mono-ADP-ribosyltransferase activity of PARP10 and facilitates HCC progression. Oncogene 39 (15), 3145–3162. 10.1038/s41388-020-1205-8 32060423

[B39] Tian Y. Korn P. Tripathi P. Komnig D. Wiemuth D. Nikouee A. (2020b). The mono-ADP-ribosyltransferase ARTD10 regulates the voltage-gated K(+) channel Kv1.1 through protein kinase C delta. BMC Biol. 18 (1), 143. 10.1186/s12915-020-00878-1 33059680 PMC7558731

[B40] Todorova T. Bock F. J. Chang P. (2014). PARP13 regulates cellular mRNA post-transcriptionally and functions as a pro-apoptotic factor by destabilizing TRAILR4 transcript. Nat. Commun. 5, 5362. 10.1038/ncomms6362 25382312 PMC4228382

[B41] Verheugd P. Forst A. H. Milke L. Herzog N. Feijs K. L. Kremmer E. (2013). Regulation of NF-κB signalling by the mono-ADP-ribosyltransferase ARTD10. Nat. Commun. 4, 1683. 10.1038/ncomms2672 23575687

[B42] Wang Y. Luo W. Wang Y. (2019). PARP-1 and its associated nucleases in DNA damage response. DNA Repair (Amst) 81, 102651. 10.1016/j.dnarep.2019.102651 31302005 PMC6764844

[B43] Wright R. H. Lioutas A. Le Dily F. Soronellas D. Pohl A. Bonet J. (2016). ADP-ribose-derived nuclear ATP synthesis by NUDIX5 is required for chromatin remodeling. Science 352 (6290), 1221–1225. 10.1126/science.aad9335 27257257

[B44] Wu C. F. Xiao M. Wang Y. L. Threadgill M. D. Li M. Tang Y. (2020). PARP10 influences the proliferation of colorectal carcinoma cells, a preliminary study. Mol. Biol. 54 (2), 252–261. 10.31857/S0026898420020184 32392194

[B45] Yu M. Schreek S. Cerni C. Schamberger C. Lesniewicz K. Poreba E. (2005). PARP-10, a novel Myc-interacting protein with poly(ADP-ribose) polymerase activity, inhibits transformation. Oncogene 24 (12), 1982–1993. 10.1038/sj.onc.1208410 15674325

[B46] Yu S. W. Wang H. Poitras M. F. Coombs C. Bowers W. J. Federoff H. J. (2002). Mediation of poly(ADP-ribose) polymerase-1-dependent cell death by apoptosis-inducing factor. Science 297 (5579), 259–263. 10.1126/science.1072221 12114629

[B47] Zhao Y. Hu X. Wei L. Song D. Wang J. You L. (2018). PARP10 suppresses tumor metastasis through regulation of Aurora A activity. Oncogene 37 (22), 2921–2935. 10.1038/s41388-018-0168-5 29515234

[B48] Zhao Y. Liang X. Wei L. Liu Y. Liu J. Feng H. (2021). RNF114 suppresses metastasis through regulation of PARP10 in cervical cancer cells. Cancer Commun. 41 (2), 187–191. 10.1002/cac2.12132 PMC7896744 33417305

