# Supplementary material for: Depletion of PARP10 inhibits the growth and metastatic potential of oral squamous cell carcinoma
Source: Front Genet. 2022 Oct 13;13:1035638. doi: 10.3389/fgene.2022.1035638 (PMC9608182; doi:10.3389/fgene.2022.1035638)
Supplement: Supplementary file 2 [file Table1.docx]

**TABLE S1** | Performance of PARP10 expression to differentiate OSCC tissues from adjacent normal tissues in GSE37991 cohort.

| Disease status | Test Positive (n) | Test Negative (n) | Total (n) | PPV | NPV | SEN | SPE |
| --- | --- | --- | --- | --- | --- | --- | --- |
| OSCC | 39 | 1 | 40 |  |  |  |  |
| Adjacent | 6 | 34 | 40 | 0.867 | 0.971 | 97.5% | 85.0% |
| Total (n) | 45 | 35 | 80 |  |  |  |  |

PPV, positive predictive value; NPV, negative predictive value; SEN, sensitivity; SPE, specificity.
