# Supplementary material for: Depletion of PARP10 inhibits the growth and metastatic potential of oral squamous cell carcinoma
Source: Front Genet. 2022 Oct 13;13:1035638. doi: 10.3389/fgene.2022.1035638 (PMC9608182; doi:10.3389/fgene.2022.1035638)
Supplement: Supplementary file 3 [file DataSheet1.pdf]

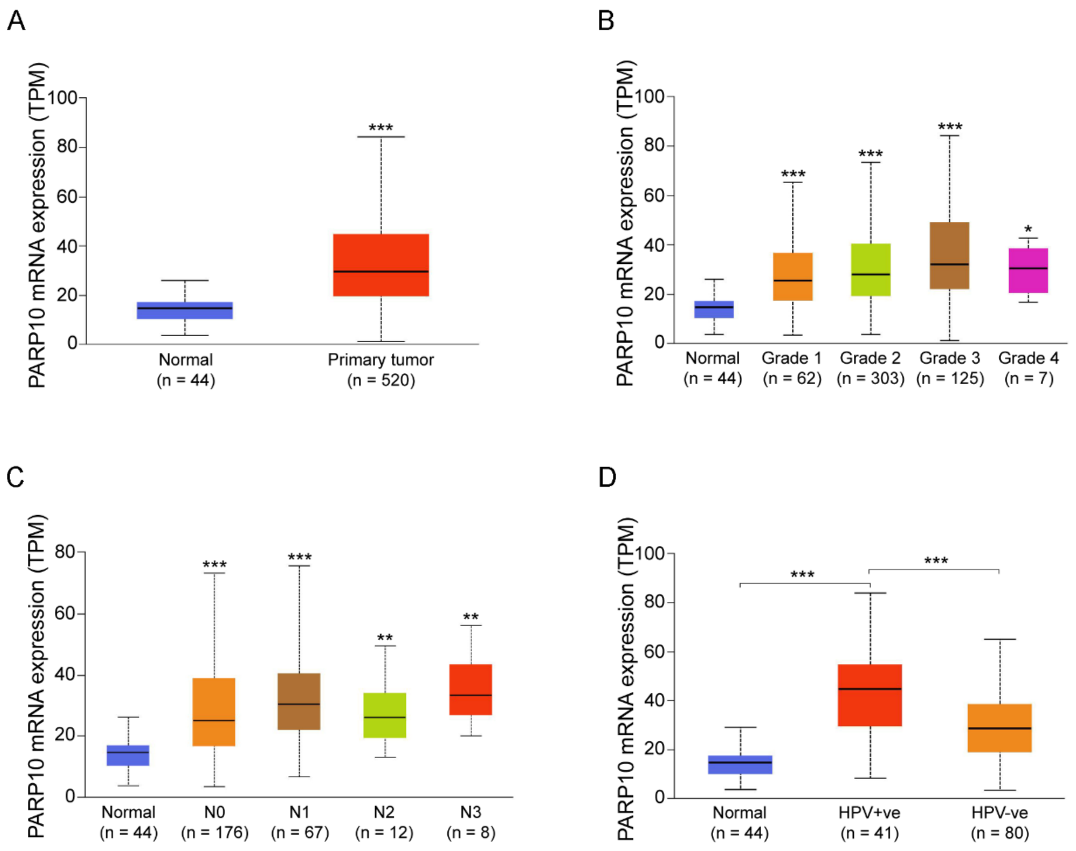

FIGURE S1 | PARP10 is elevated in HNSCC. **(A-D)** The expression levels of PARP10 in primary HNSCC tissues **(A)** and in HNSCC patients at different tumor grades **(B)**, lymph node metastasis statuses **(C)**, and HPV infection statuses **(D)** were analyzed in the UALCAN database. HNSCC, head and neck squamous cell carcinoma; TPM, transcript per million. $^{*}p < 0.05$, $^{**}p < 0.01$, $^{***}p < 0.001$.

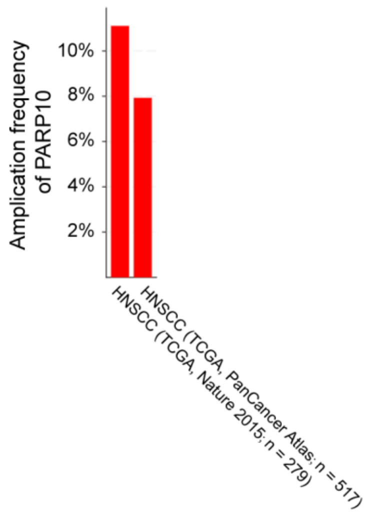

FIGURE S2 | PARP10 gene is amplified in HNSCC. The amplification frequency of the PARP10 gene in HNSCC was investigated in the cBioPortal database.

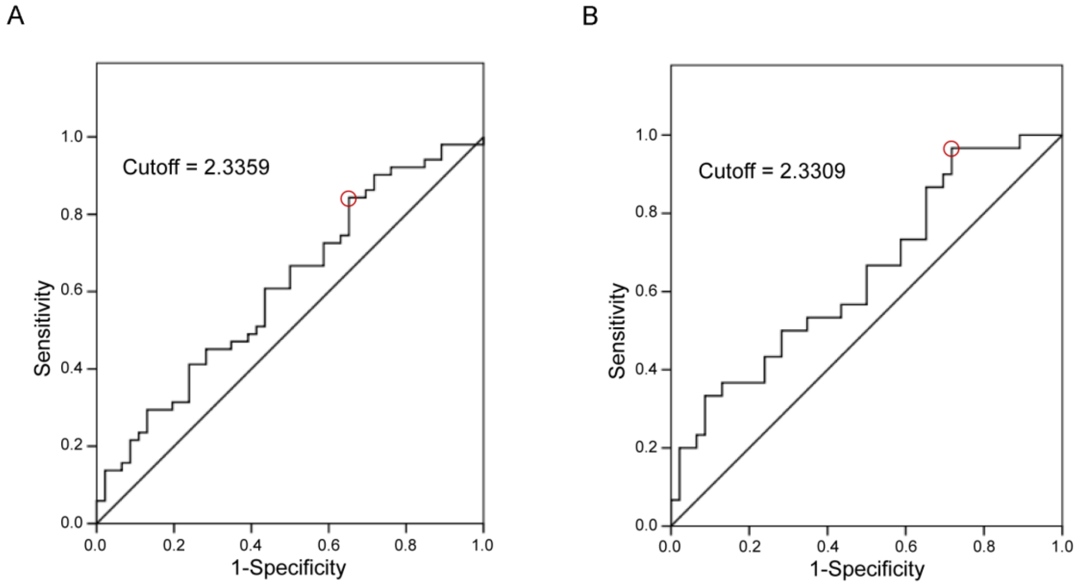

FIGURE S3 | Determination of the cutoff value for PARP10 expression. **(A**, **B)** ROC curves were applied to determine the PARP10 cutoff values for overall survival **(A)** and disease-specific survival **(B)**.

A

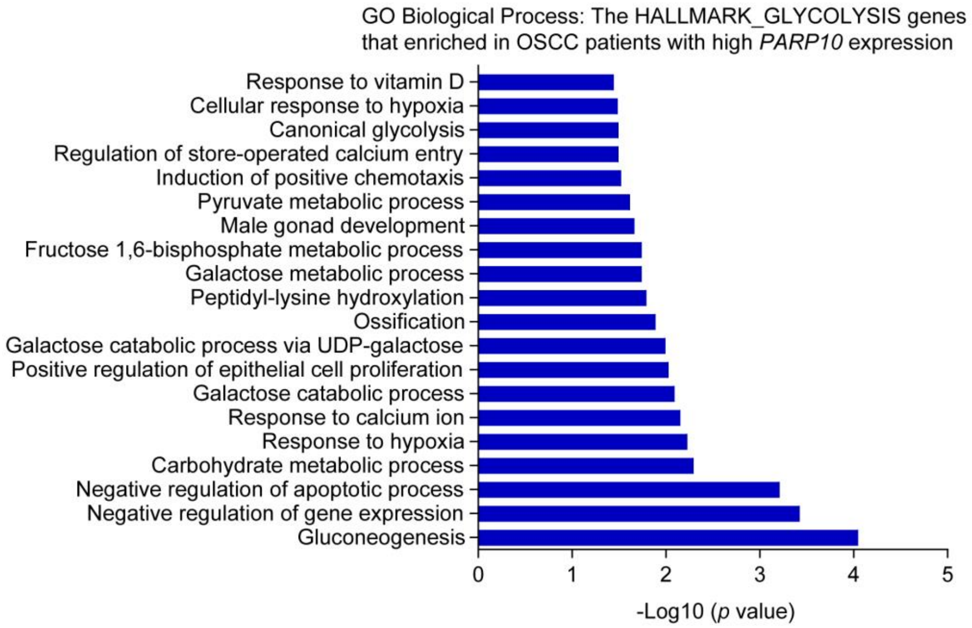

B

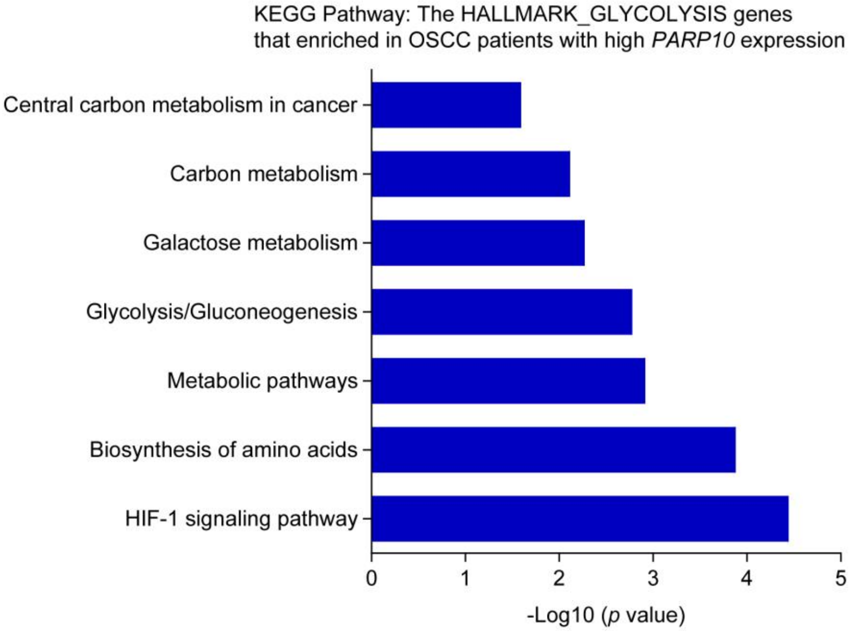

FIGURE S4 | HALLMARK_GLYCOLYSIS genes that enriched in OSCC with high *PARP10* expression are related to various metabolic processes and pathways. **(A**, **B)** The HALLMARK_GLYCOLYSIS genes that enriched in OSCC with high *PARP10* expression were subjected to GO analysis for enriching their biological functions **(A)** and pathways **(B)**.

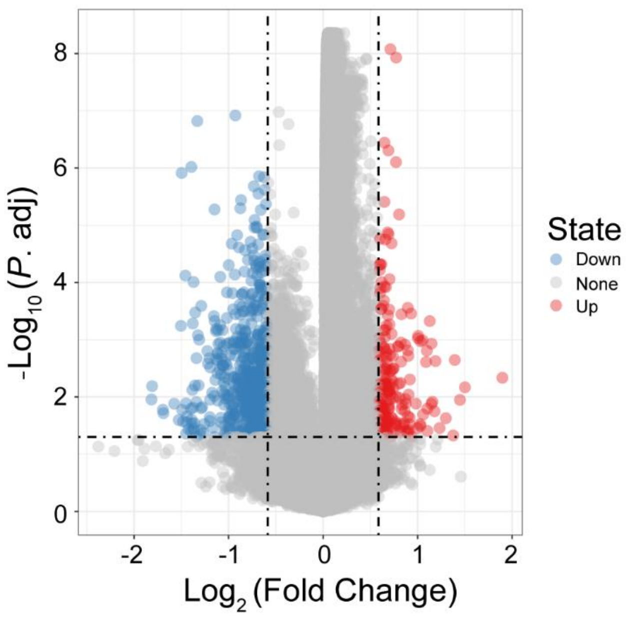

FIGURE S5 | Distribution of PARP10-related DEGs is shown in volcano plots. Volcano plots of the DEGs in the *PARP10*^{High} versus *PARP10*^{Low} group in GSE41613. DEGs, differentially expressed genes.
